# Understanding Atrial Fibrillation Complexity Through the Lens of Turbulence Dynamics: Implications for Treatment Strategies

**DOI:** 10.1111/jce.70229

**Published:** 2025-12-21

**Authors:** Xin Chu, Xiaohan Jiang, Qing Qiao, Xiaojun Wang, Hong Ye

**Affiliations:** ^1^ Department of Cardiovascular Medicine Anhui Chest Hospital Hefei Anhui China; ^2^ Anhui Provincial Center for Disease Control and Prevention Hefei Anhui China

**Keywords:** atrial electrophysiological properties, atrial fibrillation, catheter ablation, left atrial remodeling, nonlinear dynamics, turbulent‐like electrical activity

## Abstract

Atrial fibrillation (AF) is the most common sustained cardiac arrhythmia encountered in clinical practice. Its incidence increases significantly with age and has become a major global public health issue. Although research into the mechanisms of AF has spanned over a century‐ranging from the reentry theory to the rotor hypothesis‐none of these theories can fully explain its complex dynamic characteristics. The electrical activity of AF exhibits similarities to fluid turbulence, and this analogy provides a new theoretical framework for understanding AF. This review systematically outlines the evolution of AF theories and analyzes the multidimensional connections between the electrophysiological properties of atrial myocardium and the principles of turbulence dynamics. These include the nonlinear propagation characteristics of electrical wavefronts, the impact of atrial tissue heterogeneity on wave conduction, and the therapeutic rationale of catheter ablation targeting the sources of turbulence. Based on this, the study proposes the hypothesis that cardiac electrical activity in AF resembles a turbulence‐like state, suggesting that AF fundamentally represents a nonlinear dynamic turbulence‐like phenomenon of myocardial excitation waves under certain conditions. This hypothesis posits that critical arrhythmogenic substrates in AF—characterized by established structural and electrophysiological heterogeneities—create conditions analogous to sites where turbulence dynamics emerge in fluid systems, providing a phenomenological framework for characterizing the spatial‐temporal organization underlying ablation therapy efficacy. By integrating traditional AF theories with fluid dynamics concepts of turbulence, this hypothesis holds promise for more comprehensively explaining the complex characteristics and individual variability of AF, thereby offering a new theoretical foundation for improving AF management outcomes.

With accelerating global population aging and improved survival rates among chronic disease patients, the incidence and prevalence of atrial fibrillation (AF) continue to rise substantially [1]. The Framingham Heart Study demonstrates that AF prevalence has tripled over the past five decades, with projections indicating that Asia alone will harbor over 72 million AF patients by 2050 [2, 3]. Epidemiological data consistently shows that AF incidence increases markedly with advancing age [2, 3, 4]. Currently, China reports approximately 20 million AF patients [5], with prevalence reaching an alarming 5.9% among octogenarians and beyond [6]. Since 1910, investigations into AF mechanisms have primarily revolved around two major hypotheses: the “reentry” theory (multiple wavelet reentry) and the “driver” theory (focal sources with fibrillatory conduction) [7]. Contemporary AF mechanism research encompasses reentry mechanism hypotheses (including multiple wavelet reentry, leading circle reentry, and rotor mechanisms) and triggered activity hypotheses [8], though significant controversies persist regarding these theoretical frameworks [9, 10].

Notably, the electrical activity in AF exhibits remarkable similarities to fluid turbulence, including irregular conduction patterns, hypersensitivity to triggering events [11], and dependence on structural boundary conditions [12]. These parallels suggest that turbulence theory may provide novel insights into understanding the complex nature of AF [13]. Turbulence theory—a branch of physics investigating random, chaotic fluid motion under high Reynolds number conditions—characterizes phenomena such as vortex formation, multi‐scale pattern organization, and self‐sustaining dynamics that may provide conceptual insights into the complex electrical conduction patterns and maintenance mechanisms in AF. The distinctive electrophysiological properties of atrial myocardium, including heterogeneous conduction velocities, diverse reentrant pathways, and variable refractory periods, render it an ideal substrate for turbulence‐like electrical activity propagation. This review aims to systematically analyze the relationship between atrial myocardial electrophysiological characteristics and AF pathogenesis, beginning with classical electrophysiological theories and exploring the potential value of electrical turbulence hypotheses in explaining AF mechanisms and catheter ablation efficacy. The insights presented herein may provide new theoretical foundations for AF prevention and precision‐targeted therapeutic interventions.

## 1 Historical Evolution and Development of Atrial Fibrillation Theory

The genesis of AF theory dates back to the early 20th century when Mines et al. [14] pioneered the groundbreaking concept of anatomically‐based reentrant activation mechanisms in 1913. Shortly thereafter in 1914, Garrey [15] demonstrated through turtle heart experiments that conduction block could potentially induce circular propagation within myocardial tissue, validating the possibility of anatomical reentry and providing experimental substantiation for Mines' hypothesis. These seminal discoveries were subsequently synthesized by Lewis [16] into the “circular excitation hypothesis of reentry,” which postulated that reentrant activation circulating around anatomical obstacles could manifest as atrial flutter or fibrillatory electrical activity depending on the differential between the reentrant circuit dimensions and tissue refractoriness.

A pivotal shift in AF research trajectory occurred in 1949 when Scherf et al. [17] successfully induced focal activation leading to atrial tachycardia or fibrillation by applying minute quantities of aconitine to the epicardium of canine right atrial appendages, thereby establishing the foundation for the automaticity theory. Subsequently in 1959, Moe et al. [18] proposed the profoundly influential multiple wavelet hypothesis, which posited that AF maintenance necessitated the simultaneous presence of 15–30 wavelets, a concept later validated through computational modeling [19]. This theory underwent further refinement in 1985 when Allessie et al. [20] demonstrated through in vivo canine studies that AF perpetuation actually required only 4–6 wavelets. Wang et al. [21] discovered that class IC antiarrhythmic agents could suppress AF by reducing wavelet quantity, garnering widespread acceptance for the multiple wavelet hypothesis [22, 23], though the precise electrophysiological mechanisms underlying wavelet formation and maintenance remained incompletely elucidated at that time.

The discovery of functional reentry mechanisms marked another significant advancement in AF theory. In 1973, Allessie et al. [24] confirmed the existence of functional reentry independent of anatomical barriers through rabbit atrial experiments, leading to the formulation of the leading circle hypothesis. This theory proposed that such reentrant circuits operate without requiring anatomical obstacles, and that shorter wavelengths and larger atrial dimensions permit more concurrent stable leading reentrant circuits, which collectively form the substrate for AF maintenance. However, subsequent investigations revealed that AF electrical activity suppression does not entirely depend on wavelength abbreviation [25], prompting researchers to explore more precise mechanisms. The deepening investigation into functional reentry ultimately culminated in the conceptualization of spiral waves. In 1990, researchers first identified spiral wave phenomena in isolated ovine hearts, demonstrating that rapidly migrating spiral waves could induce ventricular fibrillation [26, 27]. Although initial synchronous activation mapping failed to capture stable spiral‐type activations in isolated sheep AF models, this limitation was overcome through optical mapping techniques. In 1990, Davidenko et al. [28] provided the first experimental evidence for the presence of spiral waves (rotors) in isolated sheep myocardial slices, a finding subsequently corroborated by animal cardiac modeling and optical mapping data. In 1998, Gray et al. [29] utilized optical mapping technology to record rotor‐like spiral waves triggered by heterogeneity in tissue conduction properties and refractory periods, furnishing direct evidence for the role of rotor mechanisms in AF initiation and perpetuation.

Nevertheless, existing AF pathogenesis theories‐whether rotor‐based or trigger‐based‐fail to systematically elucidate the mechanisms underlying AF initiation and cannot adequately explain why left atrial pulmonary vein isolation proves effective in treating paroxysmal AF patients. The critical role of pulmonary veins in AF pathophysiology remains insufficiently explained by current paradigms. This review aims to propose a novel turbulence‐like theory of AF pathogenesis, articulating the relevant mechanisms and interrelationships from physiological, pathological, and physical perspectives.

## 2 The Proposal and Clinical Significance of Cardiac Electrical Activity Turbulence Hypothesis

### 2.1 Theoretical Foundation and Characteristics of Electrical Activity Turbulence Hypothesis

At the microscopic level, electrical signal conduction in cardiomyocytes depends on transmembrane ionic flux. During excitation propagation, the primary driving force is an electrochemical gradient‐extracellular positive, intracellular negative‐that facilitates ionic influx, triggering cardiomyocyte depolarization and subsequent contraction. Once generated, the action potential does not remain localized to the stimulated membrane region but rapidly propagates in all directions along the membrane until it encompasses the entire cell, maintaining consistent amplitude and waveform throughout propagation. This normal physiological conduction bears certain similarities to laminar flow in fluid dynamics. The ionic properties themselves support this comparison: ions, as charged particles, constitute an integral component of fluid media in solution. Ionic movement within biological solutions (cytoplasm, extracellular fluid) conforms to core fluid definitions: the intra‐ and extracellular environments are fundamentally electrolyte solutions (liquid fluids) composed of water molecules, ions, proteins, and other constituents possessing fluidity, incompressibility, and viscosity. At the microscopic particle level, ions (such as Na⁺, K⁺, Ca²⁺) are charged particles in solution whose diffusion or directional movement essentially represents solute (electron) flow in fluid media driven by both concentration and voltage gradients. The atrial myocardium comprises a highly structured, discrete cellular lattice where individual cardiomyocytes maintain fixed spatial positions within an organized three‐dimensional architecture. Electrical coupling occurs through localized gap junction channels (primarily connexin‐40 and connexin‐43) concentrated at intercalated discs, creating discrete resistive pathways rather than continuous conductivity [30, 31]. This lattice exhibits pronounced structural anisotropy: longitudinal conduction velocity along aligned myocardial fiber directions exceeds transverse conduction velocity by 2‐3 fold due to preferential gap junction distribution and fiber geometry [30].

Notably, hundreds of millions of cardiomyocytes are coupled via gap junctions to form a functional syncytium, integrating individual cellular ionic currents into collective behavior resembling fluid flow at the macroscopic scale. Critically, this phenomenological similarity describes spatiotemporal pattern organization rather than transport mechanisms: cardiac excitation propagates through sequential local depolarizations where stationary cardiomyocytes within a fixed myocardial lattice undergo state changes (excited vs. refractory), whereas fluid turbulence involves advective mass transport where fluid particles physically displace carrying momentum and energy. Electrical wave propagation follows reaction‐diffusion dynamics governed by ion channel kinetics and intercellular current diffusion, fundamentally distinct from Navier‐Stokes equations governing fluid momentum advection. According to Boron & Boulpaep Medical Physiology, ordinary atrial myocardium exhibits relatively slow conduction velocities of approximately 0.4 m/s. The atria contain preferential conduction pathways with aligned myocardial fibers, such as Bachmann's bundle of the anterior interatrial tract, which demonstrate accelerated conduction velocities ranging from 1.0 to 1.2 m/s.

**Figure 1:**
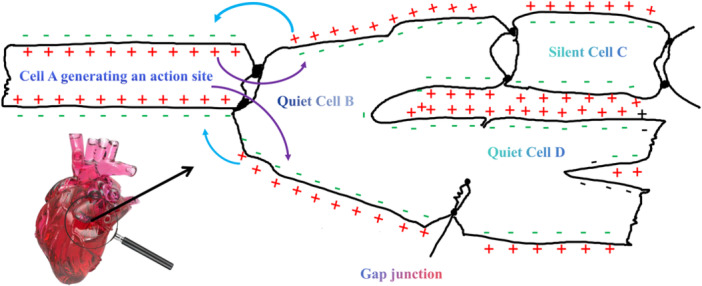
Local current flow and cell‐to‐cell conduction of cardiomyocyte action potentials. The figure illustrates the mechanism of local current flow and intercellular conduction in cardiac myocytes. When Cell A generates an action potential, the intracellular space becomes negatively charged (represented by “−” symbols) while the extracellular space accumulates positive charges (represented by “+” symbols). This electrochemical gradient drives local current flow through gap junctions to adjacent resting cells (Quiet Cell B, Silent Cells C and D). The blue curved arrows indicate the direction of intracellular depolarizing current propagation, while the external positive charges form return current pathways. Gap junctions, composed of connexin proteins, provide low‐resistance electrical coupling between cells, enabling rapid synchronous activation across the myocardium. The heart illustration (bottom left) indicates the anatomical origin of these cardiomyocytes. This ordered propagation pattern maintains uniform conduction velocity and represents the fundamental basis of normal cardiac electrical activity.

The ionic flow across cell membranes demonstrates superficial mathematical analogies to classical fluid dynamics at the microscopic scale, yet excitation propagation between cardiomyocytes fundamentally differs from fluid advection: it represents regenerative wave propagation in a discrete lattice of stationary cells governed by reaction‐diffusion equations (coupling local ion channel dynamics with intercellular current diffusion via gap junctions), whereas fluid turbulence involves continuous mass advection governed by Navier‐Stokes momentum equations. These phenomenological pattern similarities—rather than mechanistic equivalence—provide the conceptual foundation for the intercellular electrical activity turbulence analogy (Figure 1), emphasizing spatial‐temporal organization patterns while recognizing fundamental transport mechanism distinctions. Our framework builds upon the established spiral wave dynamics paradigm in excitable media—where stable spirals correspond to tachycardia and spiral wave breakup constitutes fibrillation's mechanistic basis—by borrowing turbulence phenomenology to characterize multi‐scale hierarchical organization and cross‐scale energy cascade patterns that complement mesoscopic spiral wave mechanistic explanations.

### 2.2 Distinction Between Rotor Fragmentation and Turbulent Electrical Activity: A Pragmatic Perspective

From a clinical and mechanistic standpoint, it is essential to distinguish between rotor fragmentation and turbulent electrical activity, despite their superficial similarities in mapping presentations. Rotor fragmentation refers to the breakdown of organized rotational activation patterns (rotors or spiral waves) into multiple smaller, irregular wavelets—a deterministic process rooted in traditional reentry theory where a stable parent rotor undergoes tip meandering and eventual break‐up when encountering heterogeneous tissue substrates [32, 33]. In contrast, turbulent electrical activity represents a fundamentally different state: a self‐sustaining, multi‐scale chaotic phenomenon governed by nonlinear dynamics principles, where local vortical structures continuously form, interact, and dissipate without requiring stable mother rotors [34, 35]. Key pragmatic distinctions include: (1) Temporal stability: rotors maintain identifiable phase singularities persisting for multiple cycles (≥ 2 rotations) before fragmenting, whereas turbulent activity exhibits phase singularities appearing and disappearing within single cycles with quasi‐random spatial migration [36, 37]; (2) Mechanistic drivers: rotor fragmentation is a reactive process resulting from wave‐tissue interactions with anatomical obstacles, while turbulent activity is intrinsically generative, arising from collective nonlinear interactions creating a self‐perpetuating state [38, 39]; (3) Clinical mapping: rotor fragmentation shows gradually increasing Shannon entropy with traceable rotor core instability, whereas turbulent activity manifests persistently high Shannon entropy (> 3.5), elevated complex fractionated electrogram burden (> 40% of left atrial surface), and continuously shifting phase singularities with lifetimes < 200 ms [40, 41]; (4) Therapeutic implications: rotor‐driven AF may respond to focal ablation targeting stable cores, while turbulent activity requires substrate modification strategies such as pulmonary vein isolation combined with posterior wall isolation to reduce the critical mass supporting turbulence maintenance [42, 43]. In summary, rotor fragmentation represents a transitional phase within the reentry paradigm, whereas turbulent electrical activity constitutes a fundamentally different electrophysiological state characterized by emergent complexity arising from multi‐scale nonlinear interactions [44]. This distinction is critical for interpreting mapping data, understanding ablation mechanisms, and developing targeted therapeutic strategies.

### 2.3 Multi‐Scale Wavefront Fragmentation: From Macro‐Scale Coherent Activation to Micro‐Scale Wavelets

**Figure 2:**
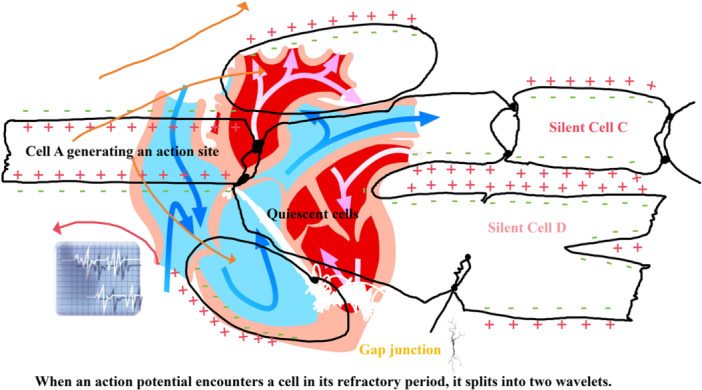
Shunting effect of refractory myocardial cells on action potential propagation. The figure demonstrates the wavefront splitting phenomenon when propagating action potentials encounter cells in their refractory period. The left panel shows a unified wavefront originating from Cell A (generating an action potential site). When this wavefront encounters the quiescent cell (center) that is still in its refractory period (indicated by the darkened region with reduced excitability), the electrical impulse cannot propagate directly through this “functional obstacle.” Instead, the wavefront bifurcates into two daughter wavelets (blue and red curved arrows), propagating around the refractory cell through alternative pathways to reach Silent Cells C and D. The heart illustration with superimposed electrical trace (left) indicates the temporal relationship between action potential phases and refractoriness. The orange background gradient and blue flow patterns visualize the spatial redistribution of electrical energy. This splitting mechanism represents a critical precursor to reentrant arrhythmias when refractory heterogeneity becomes pronounced in diseased myocardium.

The myocardial refractory period refers to the characteristic interval following cardiomyocyte excitation (action potential) during which cells cannot be re‐excited (effective refractory period, ERP) or require stronger stimulation for excitation (relative refractory period, RRP). This property fundamentally prevents cardiac tetanus, ensuring rhythmic contraction and relaxation cycles. However, in tachyarrhythmias such as AF, it becomes a critical element in the formation and maintenance of reentrant circuits. Myocardial refractory periods and their spatial heterogeneity create dynamic conduction constraints for electrical signal propagation, potentially fragmenting otherwise regular conduction wavefronts into multiple daughter wavelets. Importantly, refractoriness differs fundamentally from static physical obstacles in fluid systems: it represents a transient, state‐dependent property of the excitable medium itself, created by the propagating wave and traveling as a refractory “tail” behind the wavefront. This dynamic unexcitability—governed by ion channel recovery kinetics and action potential duration restitution properties—determines wavefront‐obstacle interactions in reaction‐diffusion systems, contrasting with mechanical barrier effects in fluid advection. Simultaneously, temporal dynamic variations in refractory periods cause these wavelets to continuously encounter new “barriers” during propagation, further modifying electrical conduction properties (Figure 2) [45].

Cardiac electrical propagation differs fundamentally from classical fluid turbulence in energy dynamics. In Kolmogorov turbulence theory, mechanical kinetic energy undergoes directional transfer from large eddies to progressively smaller eddies through nonlinear interactions, maintaining constant energy flux across the inertial subrange until ultimate conversion to heat through viscous dissipation at the Kolmogorov microscale. Cardiac electrical propagation follows entirely different physics: each wavefront excitation depends on local metabolic activity of cardiomyocyte Na⁺/K⁺‐ATPase and Ca²⁺‐ATPase pumps, with each depolarization‐repolarization cycle consuming local ATP reserves that are immediately replenished through mitochondrial oxidative phosphorylation [46, 47]. No directional energy flow or transfer occurs between spatial scales. The term “multi‐scale organization” used in this manuscript refers to geometric patterns of wavefront spatial fragmentation rather than energy cascade in the Kolmogorov sense.

Multi‐scale wavefront fragmentation in AF exhibits the following spatial organization characteristics: Large‐scale coherent wavefronts originating from pulmonary veins encounter spatial heterogeneity in atrial substrate during chamber‐level propagation—conduction velocity exhibits marked regional variations, refractory periods display spatial gradients, and anatomical structures create conduction barriers (trabeculated muscles, venous orifices). These factors cause coherent wavefront fragmentation into regional daughter waves. Daughter waves encountering further tissue heterogeneity and dynamically changing refractory period distributions undergo continued fragmentation into local wavelets, manifesting as complex fractionated electrograms and rapid local cycle lengths [46, 47]. This hierarchical fragmentation process is governed by wave‐tissue interaction physics—conduction block at refractory boundaries, wavefront curvature effects determining propagation success, and source‐sink mismatch at tissue interface transitions—rather than energy transfer mechanisms. Each wavelet formation and propagation event requires local ATP consumption by Na⁺/K⁺‐ATPase to restore transmembrane ionic gradients dissipated during action potentials, fundamentally distinguishing cardiac excitation from mechanical energy systems where daughter structures inherit kinetic energy from parent structures. Clinically, this multi‐scale spatial organization framework manifests as progressive dominant frequency gradients across atrial regions, accompanied by increasing Shannon entropy and phase singularity density from pulmonary vein sources toward peripheral atrial tissue [48]. The framework provides organizational principles for hierarchical ablation strategies: eliminating fragmentation initiators through pulmonary vein isolation, modifying substrate that amplifies fragmentation through posterior wall isolation, and targeting sites of persistent micro‐scale activity through complex fractionated electrogram ablation.

Based on these physiological characteristics, the electrical activity turbulence hypothesis draws conceptual parallels from fluid dynamics to provide an organizational framework for understanding complex electrophysiological mechanisms underlying AF. Fluid turbulence—a classical theory in fluid dynamics describing irregular, chaotic flow—exhibits nonlinear dynamic behavior, multi‐scale spatial pattern organization, hypersensitivity to initial conditions, and dependence on system boundary conditions [10]. As investigations into AF electrophysiological mechanisms have progressed, remarkable similarities have emerged between intracellular ions as charged particles in solution and macroscopic fluid flow patterns. When integrated across countless intercellular ionic currents, these phenomena constitute macroscopic electrical activity amenable to description via turbulence‐like theory [11].

This hypothesis posits that AF essentially represents a turbulence‐like state of myocardial electrical activity‐a nonlinear dynamic phenomenon manifested when atrial electrical activation waves fulfill specific conditions. Similar to fluid turbulence, electrical turbulence in AF presents as a multi‐scale chaotic process in both temporal and spatial domains, characterized by rapidly changing rotational activation patterns and irregular conduction pathways. AF electrical activity demonstrates striking similarities to fluid turbulence, including irregularity in conduction patterns, hypersensitivity to triggering events, dependence on cardiac architecture (particularly left atrial dimensions and morphology), and energy transfer characteristics across various spatiotemporal scales [12]. In this turbulence‐like state, atrial electrical activation waves undergo splitting, fragmentation, and reorganization, forming vortical structures of varying dimensions that superficially resemble rotors or wavelets described in traditional reentry theories but differ fundamentally in their physical nature and dynamic properties [8].

Notably, electrical activity turbulence represents not merely simple chaos but rather a complex state governed by specific physical principles, encompassing multilevel interactions ranging from macroscopic tissue architecture to microscopic cellular electrophysiological properties [30]. These multi‐scale interactions form the foundation for AF initiation, maintenance, and recurrence. Catheter ablation therapy effectively disrupts the turbulence state's self‐sustaining mechanisms by destroying the conditions necessary for vortex formation, with clinical efficacy indirectly validating this hypothesis. The electrical activity turbulence hypothesis transcends limitations of conventional reentry theories by reconceptualizing AF as a dynamic, self‐organizing complex system phenomenon. This paradigm helps explain the clinical heterogeneity of AF and differential therapeutic responses, providing novel perspectives for understanding AF heterogeneity and individualized treatment strategies.

**Figure 3:**
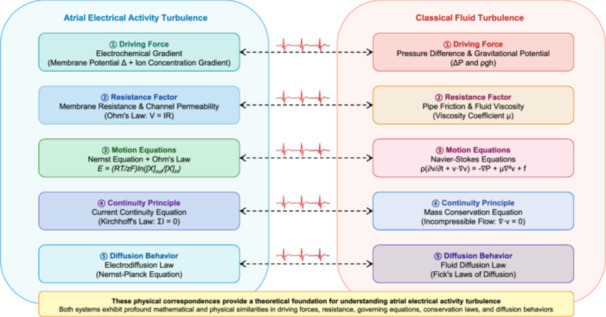
Correspondence between atrial electrical activity turbulence and classical fluid flow. The figure presents a systematic comparison between atrial electrical activity turbulence (left panel, blue background) and classical fluid turbulence (right panel, pink background), demonstrating five levels of physical correspondence. ➀ Driving Force: Electrochemical gradients (membrane potential differences+ion concentration gradients) in electrical systems correspond to pressure differences and gravitational potential energy (ΔP and ρgh) in fluid systems. ➁ Resistance Factor: Membrane resistance and ion channel permeability (governed by Ohm's Law: V = IR) in cardiac tissue correspond to pipe friction and fluid viscosity (viscosity coefficient μ) in fluid dynamics. ➂ Motion Equations: The Nernst equation combined with Ohm's Law [E = (RT/zF)ln([X]out/[X]in)] governs ionic current flow, analogous to the Navier‐Stokes equations [ρ(∂v/∂t + v·∇v)=‐∇P+μ∇²v + f] governing fluid motion. ➃ Continuity Principle: Current continuity equations based on Kirchhoff's Law (ΣI = 0) in electrical circuits parallel mass conservation equations for incompressible flows (∇·v = 0). ➄ Diffusion Behavior: Electrodiffusion governed by the Nernst‐Planck equation in cardiac tissue corresponds to fluid diffusion described by Fick's Laws. The central dashed arrows with ECG‐like waveforms symbolize the mathematical and physical similarities bridging these two systems. The yellow box at the bottom emphasizes that these correspondences provide a theoretical foundation for understanding atrial electrical turbulence through established fluid mechanics principles, particularly regarding driving forces, resistance, governing equations, conservation laws, and diffusion behaviors.

As shown in Figure 3, regarding driving forces, electrical activity turbulence is propelled by electrochemical gradients (voltage differentials plus concentration gradients), while fluid turbulence is driven by pressure differentials and gravitational energy gradients. In terms of resistance factors, electrical activity turbulence is constrained by membrane resistance and channel protein permeability, analogous to the effects of pipe friction and fluid viscosity in hydrodynamic systems. The governing equations differ yet maintain conceptual parallels: electrical activity follows the Nernst equation and Ohm's law, while fluids adhere to the Navier‐Stokes equations. Regarding continuity principles, electrical activity satisfies current continuity equations (Kirchhoff's laws), while fluids obey mass conservation equations. Both systems demonstrate comparable diffusion behaviors, each following its respective diffusion laws. These correspondences establish the theoretical foundation for understanding atrial electrical activity turbulence.

From a spatial organization perspective, atrial electrical activity during AF exhibits hierarchical wavefront fragmentation from macro‐scale to micro‐scale—phenomenologically resembling multi‐scale vortical structures observed in fluid turbulence while differing fundamentally in underlying physics. Within the atria, large‐scale electrical activation waves decompose into smaller‐scale wavelets through structural heterogeneity, electrophysiological disparities, dynamic refractoriness, and wave‐tissue interaction physics, forming spatial patterns that superficially parallel scale‐dependent organization in turbulent flows [45]. This fragmentation process sustains not through inter‐scale energy transfer but through continuous local metabolic regeneration at each spatial scale via ATP‐dependent ion pumps. The primary energy expenditure in AF is metabolic rather than mechanical: ATP is consumed by transmembrane ion pumps (Na⁺/K⁺‐ATPase, SERCA2A) to restore electrochemical gradients for sodium, potassium, and calcium ions that are dissipated with each action potential. The four‐ to six‐fold acceleration in atrial activation rates during AF dramatically increases this metabolic burden, representing biochemical costs of maintaining ionic homeostasis rather than viscous dissipation of kinetic energy into heat through friction. This multi‐scale wavefront organization enables AF to manifest self‐sustaining complexity at microscopic levels while exhibiting quasi‐random characteristics macroscopically, explaining the persistent irregular rhythm observed clinically. Compared to conventional AF mechanism theories, the electrical activity turbulence theory offers superior integrative capacity and explanatory power.

Traditional reentry mechanism hypotheses (multiple wavelet reentry, leading circle reentry, and rotor mechanisms) primarily focus on specific electrophysiological phenomena and struggle to comprehensively explain the complex dynamic properties of AF [8]. In contrast, this hypothesis, by introducing turbulence concepts from fluid dynamics, provides a unified theoretical framework that better elucidates various phenomena observed in AF, including rotor formation and dissolution, wavefront fragmentation and fusion, rapid alterations in electrical activity, and sensitive dependence on initial conditions. Furthermore, this hypothesis explains the limited efficacy of conventional antiarrhythmic pharmacotherapy, as these agents typically target specific ion channels without addressing the complex systemic behavior in turbulence‐like states.

### 2.4 Electrical Activity Turbulence Hypothesis Explaining the Efficacy of AF Ablation Therapy

This hypothesis offers a novel perspective for understanding the efficacy of AF catheter ablation, particularly pulmonary vein isolation. From a turbulence theory standpoint, the pulmonary vein‐left atrial junction can be conceptualized as a “turbulence generator” within the cardiac electrical activity system—a critical structure for producing and maintaining electrical activity turbulence. This region possesses ideal conditions for turbulence formation: myocardial sleeve heterogeneity, complex geometric architecture, localized variations in electrophysiological properties, and “impedance mismatch” with the left atrial myocardium proper.

The electrical activity turbulence hypothesis explains AF triggering and maintenance through the lens of vortex generation and propagation. Turbulence generation exhibits specific spatial and temporal characteristics: spatially manifesting as vortex formation and propagation; temporally presenting as similar but non‐identical periodic behavior, quantifiable through assessment of adjacent cardiac cycle similarity (approximate entropy in nonlinear dynamics). Research demonstrates that atrial electrical activity in AF patients maintains irregularity even after double filtering‐behavioral characteristics of “physiological noise” consistent with deterministic chaotic systems, supporting the conceptualization of AF as a nonlinear dynamic phenomenon.

**Figure 4:**
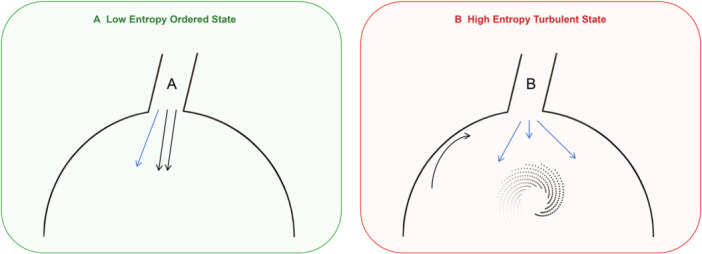
Comparison of myocardial electrical activity vortex formation patterns under different triggering mechanisms. The figure illustrates two contrasting patterns of electrical wave propagation and their relationship to turbulence generation. Panel A (Low Entropy Ordered State): The left panel, outlined in green, shows a simplified hemispherical representation of cardiac tissue. Two parallel blue arrows descend vertically and uniformly, labeled “A,” indicating organized, unidirectional wavefront propagation. This pattern represents an ordered activation sequence with low spatial complexity. Panel B (High Entropy Turbulent State): The right panel, outlined in red, shows the same hemispherical tissue geometry but with markedly different activation dynamics. The initial impulse (labeled “B”) splits into multiple divergent blue arrows that propagate in different directions. Within the tissue, a spiral pattern (shown as a dotted vortex) emerges, representing rotational electrical activity and phase singularity formation. This pattern illustrates wavefront fragmentation and chaotic propagation associated with high entropy states.

Pulmonary vein isolation proves effective because it electrically disconnects this critical arrhythmogenic substrate through established electrophysiological mechanisms: isolating primary ectopic trigger sources and substrate critical for reentry initiation and maintenance. By creating circumferential ablation lines at the pulmonary vein‐left atrial junction, the procedure effectively blocks: (1) Ectopic trigger propagation: automaticity within pulmonary vein myocardial sleeves cannot propagate to the left atrial myocardium proper, even if present; (2) Critical substrate for reentry: eliminating conduction heterogeneity and functional reentrant circuits at the pulmonary vein‐left atrial junction—structures that foster local micro‐reentry and wavefront fragmentation; (3) Trigger‐substrate interaction pathways: severing conduction pathways through which pulmonary vein triggers interact with heterogeneous atrial substrate while eliminating key structural discontinuities capable of sustaining complex wavefront dynamics (Figure 4).

From a systems dynamics perspective, pulmonary vein isolation effectively removes key driving factors that maintain the system in a high‐entropy (turbulence‐like) state, facilitating its return to a low‐entropy (ordered) state. This understanding aligns with findings by Vogl et al. [31], demonstrating that catheter ablation significantly reduces electrical activity turbulence characteristics by altering left atrial conduction patterns. The electrical activity turbulence hypothesis further explains why some complex AF patients require additional ablation strategies. In these patients, the atrial myocardium proper may have undergone extensive structural and electrical remodeling, forming multiple secondary “turbulence maintenance points” such as rotational electrical activity anchoring zones or high‐incidence wave fragmentation regions. Under these circumstances, pulmonary vein isolation alone may insufficiently eliminate all turbulence sources, necessitating supplementary substrate ablation (linear ablation, complex fractionated electrogram ablation, or vortex center ablation) to further reduce atrial turbulence propensity.

This hypothesis also elucidates mechanisms of AF recurrence following catheter ablation. If gaps exist in ablation lines or conduction recovers, the original “turbulence generator” may reactivate; concurrently, even with complete pulmonary vein isolation, newly developed structural and electrophysiological heterogeneities within the atrial myocardium may form new “turbulence generation points,” triggering AF recurrence. This corresponds with clinical observations: early recurrence typically relates to pulmonary vein reconnection, whereas late recurrence more frequently associates with progressive atrial substrate remodeling.

In conclusion, the electrical activity turbulence hypothesis provides an integrative theoretical framework for understanding AF pathogenesis and catheter ablation efficacy. By combining traditional electrophysiological theories with nonlinear dynamics principles, it not only explains known AF electrophysiological phenomena but also furnishes new theoretical foundations for optimizing AF treatment strategies, predicting therapeutic responses, and preventing recurrence. Future research should further explore developing more precise AF classification methodologies and personalized treatment strategies based on quantitative indicators of electrical activity turbulence characteristics.

## 3 Current Research Status on Atrial Electrical Activity Turbulence Mechanisms and Their Association With Atrial Fibrillation

### 3.1 Mechanoelectrical Coupling and Transformation to Electrical Activity Turbulence: Ion Channels and Atrial Myocardial Electrophysiological Properties

While spiral wave theory rigorously describes rotor dynamics and breakup mechanisms at the mesoscopic scale through reaction‐diffusion physics, our turbulence‐inspired framework provides complementary phenomenological tools for characterizing cross‐scale statistical patterns from microscopic substrate heterogeneities to macroscopic activation disorganization. A complex interrelationship exists between atrial myocardial electrophysiological properties and AF pathogenesis. Research by Shen et al. [49] on calcium‐activated potassium channels revealed that aberrant alterations in ion channel activity can induce significant changes in atrial myocardial electrophysiological characteristics. Particularly under pathological conditions, mRNA and protein expression levels of IKCa2.3/3.1, AKT1, and P300 demonstrate marked elevation (*p* < 0.05). These alterations in ion channel expression directly modulate cardiomyocyte action potential properties, establishing a substrate conducive to atrial electrical activity disorder. These findings provide crucial experimental evidence for understanding the association between fundamental myocardial electrophysiological properties and AF development.

Non‐physiological electrical conduction patterns play pivotal roles in AF evolution. Research demonstrates that under normal physiological conditions, atrial electrical activity exhibits relatively ordered propagation patterns, whereas under pathological conditions, this electrical activity manifests conspicuous disorganization characteristics [50]. This electrical disorganization is particularly prone to formation within the complex anatomical architecture of the left atrium. High‐density electrogram technology has been validated for multifaceted capture of electrical activity patterns, such as conduction velocity, local conduction block, and quantification of rotational activity. Studies have confirmed that in AF patients, electrograms display pronounced nonlinear dynamic characteristics, manifested as elevated Shannon entropy, wavefront fragmentation, and increased phase singularities‐features highly analogous to the dynamic properties of fluid turbulence [50]. These findings establish a solid scientific foundation for the development of atrial electrical activity turbulence theoretical frameworks.

From the electrical activity turbulence perspective, these research findings reveal the transition process of atrial electrical activity from ordered to chaotic states. This distinctive electrical activity pattern demonstrates characteristics structurally and functionally similar to turbulence in fluid dynamics, potentially representing a novel approach for understanding AF initiation and maintenance mechanisms.

### 3.2 Application of Nonlinear Dynamics in Atrial Electrical Activity Turbulence Research

Nonlinear dynamics models integrated with patient‐specific electrophysiological mapping have been extensively employed to investigate the generative mechanisms of atrial electrical activity disorder [51]. We emphasize that our analogy framework explicitly acknowledges the three‐dimensional complexity of atrial myocardial architecture, including transmural fiber orientation variations, wall thickness heterogeneity, and endo‐epicardial electrophysiological gradients. Critically, phenomena such as endo‐epicardial dissociation—where electrical activity becomes spatially uncoupled between inner and outer atrial wall layers due to transmural conduction block—represent arrhythmogenic mechanisms directly arising from this structured 3D substrate [52]. Our turbulence‐inspired terminology describes emergent spatial‐temporal fragmentation patterns within this complex anisotropic geometry rather than reducing cardiac tissue to a homogeneous, isotropic medium. The phenomenological analogy serves to characterize multi‐scale disorganization patterns while recognizing that underlying propagation physics remains governed by anisotropic reaction‐diffusion dynamics in a discrete cellular lattice with deterministic structural constraints. These models elucidate the dynamic characteristics of atrial electrical activity transition from ordered to chaotic states, providing crucial support for electrical activity turbulence theory.

Dedè et al. [53] constructed an idealized atrial electrophysiological computational model to investigate complex electrical activity dynamics. This research solved reaction‐diffusion equation systems, parameterizing conduction velocity and repolarization characteristics based on electrophysiological data, while simultaneously simulating atrial myocardial‐tissue interface properties through boundary conditions. The study employed finite element methodology to simulate atrial electrical activity propagation and incorporated bifurcation theory from nonlinear dynamics to explain the transition of electrical activity from ordered to chaotic states under physiological conditions, establishing theoretical foundations for understanding AF pathogenesis. From an electrical activity turbulence perspective, this model successfully simulated disorganized electrical behavior under specific conditions.

Parker et al. [54] developed a novel multimodal electrophysiological modeling approach, integrating high‐resolution atrial anatomical reconstruction with electrophysiological property mapping. This model demonstrated electrophysiological heterogeneity across different atrial regions, with particularly significant action potential duration inconsistencies and conduction velocity variations at the pulmonary vein‐left atrial junction‐precisely the hotspots for electrical wave fragmentation and rotor formation. The research further observed significant positive correlations between left atrial volume and electrical activity complexity index (*R* ^2^ = 0.83), as well as between pulmonary vein vestibule area and rotor formation probability (*R* ^2^ = 0.70). These findings hold substantial significance within the electrical activity turbulence theoretical framework, validating the synergistic effects of atrial anatomical structures and electrophysiological properties in chaotic electrical activity formation, while providing theoretical support for understanding the efficacy of catheter ablation targeting these critical structures.

### 3.3 Characteristics and Maintenance Mechanisms of AF Electrical Activity Turbulence

Corti et al. [55] conducted in‐depth investigations into AF electrical activity characteristics, analyzing differences in left atrial electrical activity between normal rhythm and AF states. This research established models based on patient‐specific left atrial electroanatomy and examined the influence of geometric and electrophysiological features on electrical activity disorder. To quantify the degree of electrical activity chaos, researchers proposed a novel metric—“phase entropy”—capable of quantitatively describing electrical activity chaos levels and effectively distinguishing between normal conduction and disorganized electrical activity. From an electrical activity turbulence theoretical perspective, this phase entropy metric effectively quantifies electrical activity turbulence intensity, providing a quantitative assessment tool for this theory. Böck et al. [56] discovered through patient‐specific electrophysiological studies that even during sinus rhythm, paroxysmal AF patients exhibit significantly higher nonlinear characteristics in atrial electrical activity compared to control groups. This finding suggests that the atrial myocardial electrophysiological substrate in AF patients has already undergone alteration even during normal rhythm, creating conditions conducive to abnormal electrical activity triggering and maintenance. These fundamental electrophysiological property changes may represent early markers of electrical activity turbulence formation.

Scarsoglio et al. [57] pioneered the application of high‐resolution optical mapping technology combined with mathematical modeling to investigate atrial electrical activity characteristics during AF. The research explored nonlinear dynamic properties of electrical activity under various rhythm and frequency conditions, revealing that electrical activity during AF exhibits pronounced chaotic characteristics, including elevated Lyapunov exponents, expanded phase space dimensions, and reduced phase synchronicity. The study confirmed that higher‐rate AF presents more complex electrical activity disorder patterns, consistent with clinical observations that rapid AF is more resistant to spontaneous termination.

These research findings collectively establish the scientific foundation for electrical activity turbulence theory, revealing the transition process and maintenance mechanisms of AF electrical activity from ordered to chaotic states. From a fluid dynamics turbulence analogy perspective, atrial electrical activity during AF exhibits characteristics similar to classical turbulence: hierarchical spatial pattern organization across multiple scales, formation and dissipation of local vortical structures (electrical rotors), and overall system unpredictability. Importantly, multifractal analysis of high‐resolution electrical recordings from chronic AF patients reveals dynamics resembling “multifractal white noise” with uncorrelated energy magnitudes over time, distinctly lacking the slow, persistent correlations characteristic of multiplicative cascade processes in classical turbulence [58]. This empirical finding aligns with our framework's central premise that AF organization reflects geometric spatial fragmentation patterns sustained by local metabolic regeneration rather than Kolmogorov‐type energy flux between scales. This turbulence‐like theoretical framework provides novel perspectives for AF mechanism research and treatment strategy development.

### 3.4 Regulatory Effects of Catheter Ablation on Atrial Electrical Activity Turbulence

As a crucial AF treatment modality, catheter ablation mechanistically regulates atrial electrical activity by destroying key sources of electrical disorder and maintenance structures. Vogl et al. [59] investigated the impact of AF catheter ablation on atrial electrical activity, discovering that pulmonary vein isolation effectively eliminates abnormal electrical activity sources at the pulmonary vein‐left atrial junction, thereby interrupting disorganized electrical activity propagation to the atrial body. Local conduction block induced by ablation forms electrical isolation zones, effectively isolating pulmonary vein triggering foci and critical micro‐reentrant substrates in the vestibular region. This research emphasizes that catheter ablation fundamentally alters atrial electrical activity dynamic properties through physical isolation of abnormal electrical activity sources. From an electrical activity turbulence perspective, this process essentially inhibits turbulence generation and maintenance through local structural destruction.

Lin et al. [60] discovered through high‐density electrogram and phase mapping technologies that AF patients exhibit pronounced electrical activity disorder characteristics, manifested as multiple phase singularities and wavefront fragmentation. Successful AF catheter ablation significantly reduces these electrical activity disorder indicators but cannot completely eliminate nonlinear characteristics of atrial electrical activity in certain patients, potentially explaining the electrophysiological basis for AF recurrence. The research further found that post‐ablation reduction in rotor activity closely correlates with long‐term sinus rhythm maintenance, indicating that effective suppression of disorganized electrical activity constitutes a key indicator of ablation success.

From our phenomenological framework, catheter ablation regulates atrial electrical activity through multiple established mechanisms: electrically isolating the arrhythmogenic substrate at the pulmonary vein‐left atrial junction by disconnecting ectopic trigger sources and eliminating heterogeneous substrate critical for reentry maintenance; while simultaneously altering atrial electrical conduction properties by creating conduction block zones that interrupt wavefront propagation pathways sustaining complex spatial‐temporal disorganization. Within our turbulence‐inspired terminology, these mechanistic interventions can be characterized as removing critical sites where substrate heterogeneity facilitates multi‐scale pattern fragmentation and reducing the structural capacity to sustain hierarchical wavefront dynamics. This multi‐level regulation of turbulence‐like activity may constitute the electrophysiological basis for catheter ablation efficacy in treating AF. Additionally, quantifiable post‐ablation residual turbulence characteristics—including persistent phase singularities, elevated Shannon entropy, and shortened dominant frequency gradients—may serve as potential indicators for predicting AF recurrence [61, 62]. These metrics reflect incomplete suppression of the multi‐scale wavefront fragmentation cascade despite anatomical pulmonary vein isolation, suggesting persistent substrate capacity to sustain turbulence‐like electrical activity. Prospective validation of these electrophysiological markers could provide theoretical foundations for risk‐stratified follow‐up protocols and guide decisions regarding early re‐intervention versus intensified antiarrhythmic therapy in individualized treatment strategies.

To establish clinical utility, we propose three validation approaches: First, retrospective studies correlating turbulence metrics (Shannon entropy, phase singularity density) with long‐term outcomes in multi‐center ablation databases; Second, prospective trials evaluating whether intra‐procedural turbulence normalization predicts success more accurately than traditional endpoints; Third, computational modeling comparing turbulence simulations against rotor‐tracking algorithms using patient‐specific mapping data. These validation efforts are essential to determine whether this framework represents a transformative paradigm or complementary heuristic tool. Until such evidence emerges, its primary value remains conceptual rather than directive for clinical decision‐making.

Our phenomenological framework shares conceptual alignment with statistical paradigms that quantify fibrillatory complexity through stochastic formalisms. Notably, renewal theory—which models phase singularity birth and death as quasi‐stationary stochastic renewal processes—demonstrates that rotors and wavelets constitute a single biological continuum governed by statistical laws rather than requiring deterministic modeling of individual reentrant circuits [48, 63]. This statistical perspective complements our turbulence‐inspired approach: renewal theory quantifies temporal dynamics of phase singularities (creation/annihilation rates), whereas our framework characterizes spatial hierarchical organization and cross‐scale pattern cascades. Both approaches emphasize emergent statistical regularities arising from complex multi‐scale interactions rather than relying solely on deterministic mechanistic explanations. Future integration of renewal‐theoretic statistical metrics with turbulence‐inspired spatial characterization may offer comprehensive quantitative tools for post‐ablation risk stratification and recurrence prediction.

## 4 Summary

This research proposes the cardiac electrical activity turbulence state hypothesis as a novel theoretical framework for understanding the electrophysiological mechanisms of AF. This hypothesis explicitly builds upon spiral wave dynamics—the rigorous mechanistic foundation for fibrillation as spiral wave breakup in excitable media—while introducing turbulence‐inspired phenomenological terminology to characterize multi‐scale hierarchical organization. This hypothesis integrates core elements from traditional AF theories while introducing turbulence concepts from fluid dynamics, enabling better explanation of the complex dynamic characteristics and individual variations in AF. This theoretical framework constitutes a more comprehensive explanatory model for AF pathogenesis, facilitating new perspectives on AF initiation and maintenance. It should be noted that this framework operates as a phenomenological analogy rather than mechanistic identity between cardiac electrophysiology and fluid dynamics. Its clinical utility will be determined by whether turbulence‐based metrics demonstrate superior predictive capacity for therapeutic outcomes relative to traditional electrophysiological indices.

Future research should further validate and refine this hypothesis and develop diagnostic and therapeutic strategies based on turbulence‐inspired metrics to enhance precision and effectiveness in AF management. This review systematically examines the historical evolution and latest developments in AF pathogenesis mechanisms, proposing the cardiac electrical activity turbulence state hypothesis as a new phenomenological framework. Research has identified phenomenological similarities between atrial electrical activity patterns and fluid turbulence in spatial‐temporal organization and mathematical description, including nonlinear dynamic characteristics, multi‐scale pattern hierarchies, and dependence on structural boundary conditions [10, 11, 12]. Unique ion channel properties of atrial myocytes and abnormal changes in intercellular gap junctions constitute the microscopic foundation for complex wavefront dynamics. The pulmonary vein‐left atrial junction region represents a well‐established critical arrhythmogenic substrate characterized by extreme structural and electrophysiological heterogeneity—complex geometric morphology, myocardial sleeve architecture with distinctive electrophysiological properties, and substrate discontinuities prone to ectopic firing and reentry formation. Within our framework, this region serves as a critical site where these mechanistically‐defined heterogeneities create conditions conducive to initiating and sustaining multi‐scale wavefront fragmentation patterns.

Catheter ablation effectively interrupts this process by electrically isolating this arrhythmogenic substrate through established mechanisms: severing ectopic trigger propagation, eliminating heterogeneous substrate critical for reentry maintenance, and blocking trigger‐substrate interaction pathways. Within our phenomenological framework, this mechanistic efficacy can be characterized as removing critical sites facilitating multi‐scale wavefront fragmentation and reducing structural capacity to sustain hierarchical spatial‐temporal disorganization. From a nonlinear dynamics perspective, ablation reduces the energy input required for the system to maintain a turbulence‐like state, promoting its return to an ordered state [57]. This hypothesis still faces several challenges, including establishing precise quantitative indicators for electrical activity turbulence, integration with traditional AF mechanism theories, and resolution limitations of clinical electrophysiological mapping technologies. Future research directions include developing new algorithms to quantify electrical turbulence characteristics for precise AF classification, designing novel therapeutic strategies targeting key turbulence nodes, and exploring nonlinear dynamic methods to predict AF risk and treatment outcomes. The cardiac electrical activity turbulence hypothesis integrates traditional theories while introducing nonlinear dynamics principles into cardiac electrophysiology research. This approach not only deepens understanding of AF's complex dynamic properties but also establishes theoretical foundations for individualized AF treatment in the precision medicine era.

This turbulence hypothesis represents a phenomenological reinterpretation of multi‐scale atrial electrical organization rather than definitive clinical validation. The fluid dynamics analogy serves as a heuristic organizational framework for spatial patterns in atrial electrophysiology, explicitly recognizing fundamental biophysical distinctions: cardiac wavelets undergo local metabolic regeneration via ATP‐dependent ion pumps rather than receiving energy through mechanical cascade mechanisms; excitation propagates through regenerative action potentials in discrete cellular networks governed by reaction‐diffusion dynamics rather than continuous mass advection; and atrial myocardium comprises discrete, anisotropic cellular lattices with gap junction coupling rather than continuous, isotropic fluid media with molecular viscosity. While providing mechanistic insights for novel mapping strategies, definitive superiority requires prospective validation through: (1) correlating turbulence metrics (Reynolds numbers, entropy distributions) with ablation outcomes in existing datasets; (2) testing whether entropy‐based measurements predict procedural success better than conventional indices; (3) comparing turbulence‐framework simulations against rotor models using high‐density mapping data. The framework's current value lies in conceptual integration and hypothesis generation pending rigorous validation.

## Author Contributions

**Chu X:** literature review, manuscript organization, writing – original draft, writing – review and editing, validation. **Jiang XH:** conceptualization, theoretical framework development, literature synthesis, visualization, writing – original draft. **Qiao Q:** literature collection, quality control, validation, writing – review and editing. **Wang XJ:** investigation, theoretical analysis, quality control, writing – review and editing. **Ye H:** conceptualization, supervision, project administration, methodology, resources, writing – review and editing. All authors have read and agreed to the published version of this manuscript.

## Funding

The authors received no specific funding for this work.

## Ethics Statement

The authors have nothing to report.

## Consent

The authors have nothing to report.

## Conflicts of Interest

The authors declare no conflicts of interest.

## Data Availability

The authors have nothing to report.

## References

[1] G. Mingyang , H. Liu , D. Xin , et al., “China Atrial Fibrillation Epidemiology 20 Years,” Chinese Journal of Cardiology 52, no. 2 (2024): 220–226.38326077

[2] G. Lippi , F. Sanchis‐Gomar , and G. Cervellin , “Global Epidemiology of Atrial Fibrillation:An Increasing Epidemic and Public Health Challenge,” International Journal of Stroke 16, no. 2 (2021): 217–221. 31955707 10.1177/1747493019897870

[3] Z. Xia , W. Dang , X. Yang , et al., “Prevalence of Atrial Fibrillation and the Risk of Cardiovascular Mortality Among Hypertensive Elderly Population in Northeast China,” Journal of Clinical Hypertension 24, no. 5 (2022): 630–637. 35434909 10.1111/jch.14483 PMC9106073

[4] Q. Chen , Z. Yi , and J. Cheng , “Atrial Fibrillation in Aging Population,” Aging Medicine 1, no. 1 (2018): 67–74. 31942483 10.1002/agm2.12015 PMC6880740

[5] Chinese Medical Association Cardiovascular Diseases Branch, China Biomedical Engineering Society Heart Rhythm Branch ., “Guidelines for the Diagnosis and Treatment of Atrial Fibrillation in China,” Chinese Journal of Cardiology 51, no. 6 (2023): 572–618.37312479

[6] S. Shi , Y. Tang , Q. Zhao , et al., “Prevalence and Risk of Atrial Fibrillation in China:A National Cross‐Sectional Epidemiological Study,” Lancet Regional Health – Western Pacific 23 (2022): 100439. 35800039 10.1016/j.lanwpc.2022.100439 PMC9252928

[7] Hong C‐ming ., “Historical Reflections on the Pathogenesis of Atrial Fibrillation,” Medical and Philosophy (Clinical Decision Forum Edition) 32, no. 01 (2011): 44–45.

[8] X. Hongling , Z. Lu , W. Weibang , et al., “Research Progress on Rotational Mechanism and Ablation Calculation Model of Atrial Fibrillation,” Cardiovascular and Circulatory Electrophysiology 44, no. 1 (2025): 105–110, Post‐Insertion 4.

[9] Q. Mu , L. Tao , and L. Xu , “Rotational Mechanism and Mapping of Atrial Fibrillation,” Journal of Practical Electrocardiography 29, no. 5 (2020): 348–355, 362.

[10] L. Jianzhong , “Overview of Turbulence Theory by Zhou Peiyuan,” Mechanics and Practice 44, no. 05 (2022): 131–144.

[11] L. Cunjin , Y. Tingting , L. Tong , et al., “Advances in Virtual Physiological Heart Model and Atrial Fibrillation Mechanism Research,” Biochemistry and Biophysics Advances 46, no. 10 (2019): 976–992.

[12] Y. Changping , H. Running , and G. Ruyuan , “Spiral Degree: A Critical Component in Turbulence Research,” Journal of Mechanical Engineering Progress 54, no. 04 (2024): 74–112.

[13] S. M. Brown , N. K. Larsen , F. G. Thankam , and D. K. Agrawal , “Regulatory Role of Cardiomyocyte Metabolism Via AMPK Activation in Modulating Atrial Structural, Contractile, and Electrical Properties Following Atrial Fibrillation,” Canadian Journal of Physiology and Pharmacology 99, no. 1 (2021): 36–41. 33049144 10.1139/cjpp-2020-0313

[14] G. R. Mines , “On Dynamic Equilibrium in the Heart,” Journal of Physiology 46, no. 4/5 (1913): 349–383. 16993210 10.1113/jphysiol.1913.sp001596 PMC1420430

[15] W. E. Garrey , “The Nature of Fibrillary Contraction of the Heart‐Its Relation to Tissue Mass and Form,” American Journal of Physiology‐Legacy Content 33, no. 3 (1914): 397–414.

[16] T. Lewis , “Oliver‐Sharpey Lectures on the Nature of Flutter and Fibrillation of the Auricle,” BMJ 1, no. 3147 (1921): 590–593. 20770267 10.1136/bmj.1.3147.590 PMC2414985

[17] D. Scherf and R. Terranova , “Mechanism of Auricular Flutter and Fibrillation,” American Journal of Physiology‐Legacy Content 159, no. 1 (1949): 137–142. 10.1152/ajplegacy.1949.159.1.137 15391089

[18] G. K. Moe and J. A. Abildskov , “Atrial Fibrillation as a Selfsustaining Arrhythmia Independent of Focal Discharge,” American Heart Journal 58, no. 1 (1959): 59–70. 13661062 10.1016/0002-8703(59)90274-1

[19] G. K. Moe , W. C. Rheinboldt , and J. A. Abildskov , “A Computer Model of Atrial Fibrillation,” American Heart Journal 67, no. 2 (1964): 200–220. 14118488 10.1016/0002-8703(64)90371-0

[20] M. A. Allessie , W. J. E. P. Lammers , F. I. M. Bonke , et al., Experimental Evaluation of Moe's Multiple Wavelet Hypothesis of Atrial Fibrillation[M]//Zipes DP, Jalife J. Cardiac electrophysiology and arrhythmias (Grune&Straton, 1985), 265–275.

[21] Z. Wang , P. Pagé , and S. Nattel , “Mechanism of Flecainide's Antiarrhythmic Action in Experimental Atrial Fibrillation,” Circulation Research 71, no. 2 (1992): 271–287. 1628386 10.1161/01.res.71.2.271

[22] C. Kirchhof , F. Chorro , G. J. Scheffer , et al., “Regional Entrainment of Atrial Fibrillation Studied by High‐Resolution Mapping in Open‐Chest Dogs,” Circulation 88, no. 2 (1993): 736–749. 8339434 10.1161/01.cir.88.2.736

[23] J. L. Cox , T. E. Canavan , R. B. Schuessler , et al., “The Surgical Treatment of Atrial Fibrillation,” Journal of Thoracic and Cardiovascular Surgery 101, no. 3 (1991): 406–426. 1999934

[24] M. A. Allessie , F. I. M. Bonke , and F. J. G. Schopman , “Circus Movement in Rabbit Atrial Muscle as a Mechanism of Tachycardia,” Circulation Research 33, no. 1 (1973): 54–62. 4765700

[25] P. Comtois , J. Kneller , and S. Nattel , “Of Circles and Spirals:Bridging the Gap Between the Leading Circle and Spiral Wave Concepts of Cardiac Reentry,” EP Europace 7, no. Suppl 2 (2005): S10–S20. 10.1016/j.eupc.2005.05.011 16102499

[26] J. M. Davidenko , A. V. Pertsov , R. Salomonsz , W. Baxter , and J. Jalife , “Stationary and Drifting Spiral Waves of Excitation in Isolated Cardiac Muscle,” Nature 355, no. 6358 (1992): 349–351. 1731248 10.1038/355349a0

[27] R. A. Gray , J. Jalife , A. V. Panfilov , et al., “Mechanisms of Cardiac Fibrillation,” Science 270, no. 5239 (1995): 1222–1223. 7502055

[28] J. M. Davidenko , P. F. Kent , D. R. Chialvo , D. C. Michaels , and J. Jalife , “Sustained Vortex‐Like Waves in Normal Isolated Ventricular Muscle,” Proceedings of the National Academy of Sciences 87, no. 22 (1990): 8785–8789. 10.1073/pnas.87.22.8785 PMC55044 2247448

[29] R. A. Gray , A. M. Pertsov , and J. Jalife , “Spatial and Temporal Organization During Cardiac Fibrillation,” Nature 392, no. 6671 (1998): 75–78. 9510249 10.1038/32164

[30] Z. Fu , R. Dong , H. Zheng , et al., “Progress of Conductivity and Conduction Velocity Measured in Human and Animal Hearts,” Reviews in Cardiovascular Medicine 25, no. 10 (2024): 364. 39484125 10.31083/j.rcm2510364 PMC11522836

[31] L. Leybaert , M. A. J. De Smet , A. Lissoni , et al., “Connexin Hemichannels as Candidate Targets for Cardioprotective and Anti‐Arrhythmic Treatments,” Journal of Clinical Investigation 133, no. 6 (2023): e168117. 36919695 10.1172/JCI168117 PMC10014111

[32] S. M. Narayan , D. E. Krummen , K. Shivkumar , P. Clopton , W. J. Rappel , and J. M. Miller , “Treatment of Atrial Fibrillation by the Ablation of Localized Sources,” Journal of the American College of Cardiology 60, no. 7 (2012): 628–636. 22818076 10.1016/j.jacc.2012.05.022 PMC3416917

[33] J. Jalife , “Mother Rotors and Fibrillatory Conduction: A Mechanism of Atrial Fibrillation,” Cardiovascular Research 54, no. 2 (2002): 204–216. 12062327 10.1016/s0008-6363(02)00223-7

[34] S. Nattel , F. Xiong , and M. Aguilar , “Demystifying Rotors and Their Place in Clinical Translation of Atrial Fibrillation Mechanisms,” Nature Reviews Cardiology 14, no. 9 (2017): 509–520. 28383023 10.1038/nrcardio.2017.37

[35] S. V. Pandit and J. Jalife , “Rotors and the Dynamics of Cardiac Fibrillation,” Circulation Research 112, no. 5 (2013): 849–862. 23449547 10.1161/CIRCRESAHA.111.300158 PMC3650644

[36] R. A. Gray , A. M. Pertsov , and J. Jalife , “Spatial and Temporal Organization During Cardiac Fibrillation,” Nature 392, no. 6671 (1998): 75–78. 9510249 10.1038/32164

[37] M. Haissaguerre , M. Hocini , A. Denis , et al., “Driver Domains in Persistent Atrial Fibrillation,” Circulation 130, no. 7 (2014): 530–538. 25028391 10.1161/CIRCULATIONAHA.113.005421

[38] J. Christoph , M. Chebbok , C. Richter , et al., “Electromechanical Vortex Filaments During Cardiac Fibrillation,” Nature 555, no. 7698 (2018): 667–672. 29466325 10.1038/nature26001

[39] R. H. Clayton , O. Bernus , E. M. Cherry , et al., “Models of Cardiac Tissue Electrophysiology: Progress, Challenges and Open Questions,” Progress in Biophysics and Molecular Biology 104, no. 1–3 (2011): 22–48. 20553746 10.1016/j.pbiomolbio.2010.05.008

[40] K. Nademanee , J. McKenzie , E. Kosar , et al., “A New Approach for Catheter Ablation of Atrial Fibrillation: Mapping of the Electrophysiologic Substrate,” Journal of the American College of Cardiology 43, no. 11 (2004): 2044–2053. 15172410 10.1016/j.jacc.2003.12.054

[41] C. H. Roney , C. D. Cantwell , J. D. Bayer , et al., “Spatial Resolution Requirements for Accurate Identification of Drivers of Atrial Fibrillation,” Circulation: Arrhythmia and Electrophysiology 10, no. 5 (2017): e004899. 28500175 10.1161/CIRCEP.116.004899 PMC5434962

[42] A. Verma , C. Jiang , T. R. Betts , et al., “Approaches to Catheter Ablation for Persistent Atrial Fibrillation,” New England Journal of Medicine 372, no. 19 (2015): 1812–1822. 25946280 10.1056/NEJMoa1408288

[43] S. Mohanty , P. Mohanty , C. Trivedi , et al., “Long‐Term Outcome of Pulmonary Vein Isolation With and Without Focal Impulse and Rotor Modulation Mapping: Insights From a Meta‐Analysis,” Circulation: Arrhythmia and Electrophysiology 11, no. 3 (2018): e005789. 29545360 10.1161/CIRCEP.117.005789

[44] F. H. Fenton , E. M. Cherry , H. M. Hastings , and S. J. Evans , “Multiple Mechanisms of Spiral Wave Breakup in a Model of Cardiac Electrical Activity,” Chaos: An Interdisciplinary Journal of Nonlinear Science 12, no. 3 (2002): 852–892. 10.1063/1.1504242 12779613

[45] X. Mingxi , Y. Huantong , C. Chu , et al., “Arrhythmia Control Strategies Based on Cardiac Electrophysiology,” Heilongjiang Medical Journal 48, no. 16 (2024): 2037–2039.

[46] B. J. Hansen , J. Zhao , N. Li , et al., “Human Atrial Fibrillation Drivers Resolved With Integrated Functional and Structural Imaging to Benefit Clinical Mapping,” JACC: Clinical Electrophysiology 4, no. 12 (2018): 1501–1515. 30573112 10.1016/j.jacep.2018.08.024 PMC6323649

[47] Y. Lu , Y. Sun , L. Cai , et al., “Non‐Traditional Risk Factors for Atrial Fibrillation: Epidemiology, Mechanisms, and Strategies,” European Heart Journal 46, no. 9 (2025): 784–804. 39716283 10.1093/eurheartj/ehae887

[48] D. Dharmaprani , M. Schopp , P. Kuklik , et al., “Renewal Theory as a Universal Quantitative Framework to Characterize Phase Singularity Regeneration in Mammalian Cardiac Fibrillation,” Circulation: Arrhythmia and Electrophysiology 12, no. 12 (2019): e007569. 31813270 10.1161/CIRCEP.119.007569

[49] P. Shen , M. Ferdous , X. Wang , et al., “A Detailed Study to Discover the Trade Between Left Atrial Blood Flow, Expression of Calcium‐Activated Potassium Channels and Valvular Atrial Fibrillation,” Cells 11, no. 9 (2022): 1383. 35563689 10.3390/cells11091383 PMC9103658

[50] T. Sekine , M. Nakaza , M. Matsumoto , et al., “4D Flow MR Imaging of the Left Atrium:What Is Non‐Physiological Blood Flow in the Cardiac System?,” Magnetic Resonance in Medical Sciences 21, no. 2 (2022): 293–308. 35185085 10.2463/mrms.rev.2021-0137 PMC9680542

[51] H. A. Kjeldsberg , J. Sundnes , and K. Valen‐Sendstad , “A Verified and Validated Moving Domain Computational Fluid Dynamics Solver With Applications to Cardiovascular Flows,” International Journal for Numerical Methods in Biomedical Engineering 39, no. 6 (2023): e3703. 37020156 10.1002/cnm.3703

[52] C. A. J. van der Heijden , L. Aerts , S. M. Chaldoupi , et al., “Hybrid Atrial Fibrillation Ablation,” Annals of Cardiothoracic Surgery 13, no. 1 (2024): 54–70. 38380145 10.21037/acs-2023-afm-0129 PMC10875200

[53] L. Dedè , F. Menghini , and A. Quarteroni , “Computational Fluid Dynamics of Blood Flow in an Idealized Left Human Heart,” International Journal for Numerical Methods in Biomedical Engineering 37, no. 11 (2021): e3287. 31816195 10.1002/cnm.3287

[54] L. Parker , E. Bollache , S. Soulez , et al., “A Multi‐Modal Computational Fluid Dynamics Model of Left Atrial Fibrillation Haemodynamics Validated With 4D Flow MRI,” Biomechanics and Modeling in Mechanobiology 24, no. 1 (2025): 139–152. 39828784 10.1007/s10237-024-01901-y

[55] M. Corti , A. Zingaro , L. Dede’ , and A. M. Quarteroni , “Impact of Atrial Fibrillation on Left Atrium Haemodynamics: A Computational Fluid Dynamics Study,” Computers in Biology and Medicine 150 (2022): 106143. 36182758 10.1016/j.compbiomed.2022.106143

[56] S. Bäck , I. Skoda , J. Lantz , et al., “Elevated Atrial Blood Stasis in Paroxysmal Atrial Fibrillation During Sinus Rhythm: A Patient‐Specific Computational Fluid Dynamics Study,” Frontiers in Cardiovascular Medicine 10 (2023): 1219021. 37649669 10.3389/fcvm.2023.1219021 PMC10463733

[57] S. Scarsoglio , A. Saglietto , F. Tripoli , et al., “Cerebral Hemodynamics During Atrial Fibrillation: Computational Fluid Dynamics Analysis of Lenticulostriate Arteries Using 7?T High‐Resolution Magnetic Resonance Imaging,” Physics of Fluids (Woodbury, N.Y.: 1994) 34, no. 12 (2022): 121909. 36776539 10.1063/5.0129899 PMC9907777

[58] G. Attuel , E. Gerasimova‐Chechkina , F. Argoul , H. Yahia , and A. Arneodo , “Multifractal Desynchronization of the Cardiac Excitable Cell Network During Atrial Fibrillation II. Modeling,” Frontiers in Physiology 10 (2019): 480. 31105585 10.3389/fphys.2019.00480 PMC6492055

[59] B. J. Vogl , A. E. Shaer , M. Van Zyl , A. M. Killu , M. Alkhouli , and H. Hatoum , “Effect of Catheter Ablation on the Hemodynamics of the Left Atrium: Hemodynamics of Ablation,” Journal of Interventional Cardiac Electrophysiology 65, no. 1 (2022): 83–96. 35348999 10.1007/s10840-022-01191-3

[60] M. Lin , L. Hao , Y. Cao , et al., “Successful Catheter Ablation of Atrial Fibrillation Improves but Not Reverses the Abnormalities of Left Atrial Mechanics and Energy Loss,” Echocardiography 36, no. 4 (2019): 752–760. 30851136 10.1111/echo.14304

[61] A. S. Jadidi , H. Lehrmann , C. Keyl , et al., “Ablation of Persistent Atrial Fibrillation Targeting Low‐Voltage Areas With Selective Activation Characteristics,” Circulation: Arrhythmia and Electrophysiology 9, no. 3 (2016): e002962 [pii]. 26966286 10.1161/CIRCEP.115.002962

[62] C. J. Nalliah , G. R. Wong , G. Lee , et al., “Sleep Apnoea Has a Dose‐Dependent Effect on Atrial Remodelling in Paroxysmal but Not Persistent Atrial Fibrillation: A High‐Density Mapping Study,” EP Europace 23, no. 5 (2021): 691–700. 10.1093/europace/euaa275 33447844

[63] D. Dharmaprani , E. Jenkins , M. Aguilar , et al., “M/M/Infinity Birth‐Death Processes—A Quantitative Representational Framework to Summarize and Explain Phase Singularity and Wavelet Dynamics in Atrial Fibrillation,” Frontiers in Physiology 11 (2021): 616866. 33519522 10.3389/fphys.2020.616866 PMC7841497

